# *SNaPaer*: A Practical Single Nucleotide Polymorphism Multiplex Assay for Genotyping of *Pseudomonas aeruginosa*

**DOI:** 10.1371/journal.pone.0066083

**Published:** 2013-06-12

**Authors:** Nadia Eusebio, Tiago Pinheiro, Adelina A. Amorim, Fernanda Gamboa, Lucília Saraiva, Leonor Gusmão, António Amorim, Ricardo Araujo

**Affiliations:** 1 IPATIMUP, Institute of Molecular Pathology and Immunology, University of Porto, Porto, Portugal; 2 Faculty of Sciences, University of Porto, Porto, Portugal; 3 Department of Pneumology, Faculty of Medicine of the University of Porto and Hospital S. João, Porto, Portugal; 4 Serviço de Pneumologia, Hospitais Universidade Coimbra-CHUC, EPE, Coimbra, Portugal; 5 REQUIMTE, Laboratório de Microbiologia, Departamento de Ciências Biológicas, Faculdade de Farmácia, Universidade do Porto, Porto, Portugal; 6 Medical and Human Genetics Laboratory, Federal University of Pará (UFPA), Belém, Pará, Brazil; Naval Research Laboratory, United States of America

## Abstract

Multilocus sequence typing (MLST) represents the gold standard genotyping method in studies concerning microbial population structure, being particularly helpful in the detection of clonal relatedness. However, its applicability on large-scale genotyping is limited due to the high cost and time spent on the task. The selection of the most informative nucleotide positions simplifies genomic characterization of bacteria. A simple and informative multiplex, *SNaPaer* assay, was developed and genotyping of *Pseudomonas aeruginosa* was obtained after a single reaction of multiplex PCR amplification and mini-sequencing. This cost-effective technique allowed the analysis of a Portuguese set of isolates (n = 111) collected from three distinct hospitals and the genotyping data could be obtained in less than six hours. Point mutations were shown to be the most frequent event responsible for diversification of the Portuguese population sample. The Portuguese isolates corroborated the epidemic hypothesis for *P. aeruginosa* population. *SNaPaer* genotyping assay provided a discriminatory power of 0.9993 for *P. aeruginosa*, by testing in silico several hundreds of MLST profiles available online. The newly proposed assay targets less than 0.01% of the total MLST length and guarantees reproducibility, unambiguous analysis and the possibility of comparing and transferring data between different laboratories. The plasticity of the method still supports the addition of extra molecular markers targeting specific purposes/populations. *SNaPaer* can be of great value to clinical laboratories by facilitating routine genotyping of *P. aeruginosa.*

## Introduction

*Pseudomonas aeruginosa* is a versatile Gram-negative bacterium frequently found in association with animals and plants, as well as in environmental samples (air, water or soil). It can grow in a broad range of temperatures, although the optimal growth is observed at 37°C [1]. This wide ecological niche implies a high degree genomic plasticity and the presence of several adaptive mechanisms. Indeed, *P. aeruginosa* is not nutritionally demanding and grows in minimal culture medium with simple molecules or under deprived conditions [2]–[4].

This bacterium represents an opportunistic pathogen with high clinical relevance in intensive care units [5] and it is a common colonizer and infection-associated pathogen in patients with bronchiectasis, and particularly with cystic fibrosis (CF) [6]. In fact, *P. aeruginosa* is found in more than 50% of CF patients and it is associated with high morbidity and mortality [7]. The early stage of *P. aeruginosa* colonization is easily controlled with antibiotic treatment which eradicates rough and smooth primary populations [8]. Subsequent re-colonization by the same strain may reveal the appearance of multi-resistant and/or mucoid forms which are much more persistent in lungs. Mucoid forms overproduce alginate and can result from the conversion of smooth or rough colonies over 1.8 years [7]. The immune system of the patient overreacts to the bacteria and alginate works as a barrier to phagocytosis that facilitates bacterial damage of tissues and eventually destroys part of the lung. Genotype-phenotype studies revealed that the risk of *P. aeruginosa* infection in CF patients depends on the severity of the mutations in cystic fibrosis transmembrane conductance regulator (CFTR) gene [9]. Patients chronically colonized by *P. aeruginosa* were associated with poor lung function and the decline in lung function was faster in those patients with *P. aeruginosa* when compared with those colonized with other bacteria [10]. The preservation of normal lung function may require *P. aeruginosa* eradication before chronic airways colonization is established [11].

Niche adaptation may be the strongest driven force that influences the genetic diversity of *P. aeruginosa* and might occasionally cause the emergence of new genomic islands on the bacterial genome [12]. The strains causing infection in CF patients may be acquired from the environment and selective pressures may contribute to a successful and ubiquitous ‘core lineage’ within patient lungs. The characterization of a large number of clinical and environmental isolates collected worldwide confirmed an epidemic and largely diverse *P. aeruginosa* populations but reports of CF clones have not been widespread [13]. The populations of *P. aeruginosa* have been recently described as presenting a non-clonal structure with frequent occurrence of recombination events [13], [14]. The description of such diversity in *P. aeruginosa* is critical for infection control strategies and prevention of person-to-person transmission in clinical units [15], [16]. European guidelines recommend the physical isolation in clinics of patients chronically colonized with *P. aeruginosa* [17].

Multilocus sequence typing (MLST) represents the gold standard genotyping method in studies concerning microbial population structure and evolution. Additionally, MLST may be particularly important on the identification of infection sources and outbreaks, and for definition of extensive and endemic microbial populations [18]. This method was first employed in 1998 to identify virulent lineages of *Neisseria meningitidis* [19] and, since then, it has been successfully adapted to several prokaryotic and eukaryotic microorganisms. In 2004, MLST was applied to *P. aeruginosa* by Curran *et al.* [20]. At present, the information of more than 1,500 strains is freely available online at http://pubmlst.org/paeruginosa/and this number is expected to increase considerably. *P. aeruginosa* MLST presents a high discriminatory power (above 0.975), and the sequence analysis of seven housekeeping genes ensures reproducibility, unambiguous analysis and the possibility of comparing and transferring data between different laboratories [21], [22]. MLST is more helpful than pulsed-field gel electrophoresis (PFGE), random amplified polymorphic DNA (RAPD) and repetitive element palindromic PCR (Rep-PCR) for the detection of clonal relatedness by labeling more strains as unique that are comparable through a large online database [23]. RAPD and Rep-PCR have advantages of by being practical, fast, and consequently more amenable to high-throughput typing; however, both methods presented limited reproducibility and lower discriminatory power, being useful for identification of major clonal groups [23]–[25]. MLST may present limited value for recognition of recombination events as it considers polymorphisms located in a limited number of genes [23]. However MLST applicability is limited to small-sized collections due to the high cost and time required by the method. Hence, it is necessary to develop alternative methods able to facilitate such studies and to boost bacterial genotyping to allow for large-scale genotyping analysis.

Single nucleotide polymorphism (SNP) based methods have been tested for few bacteria, mainly for identification purposes [26], although occasionally they have also been used in the differentiation of specific lineages [27], [28]. This methodology is practical and sensitive to simultaneous analysis of some polymorphisms [29], [30]. It involves multiplex PCR of target genomic regions containing the polymorphisms, which are detected by mini-sequencing. The mini-sequencing is performed using a multiplex single base extension (SBE) primer strategy in the presence of fluorescent labeled dideoxy nucleoside triphosphates (ddNTPs). By employing SBE primers of different lengths (nucleotide tails can be added at 5′), this methodology allows the recognition of multiple size fragments by automated capillary electrophoresis. The result is a set of peaks with distinct colors that represent site-specific genomic variation.

Aiming to optimize a method able to overcome the limitations of MLST to genotype large numbers of bacterial isolates, in the present study we present a SNP based method, hereby designated *SNaPaer* assay, targeting a set of 23 polymorphisms located at seven genes. The standard MLST method requires sequencing of more than 3,300 nucleotides but only some of these are important for most applications (since most positions are conserved while a few others are extremely variable). The selection of the most informative nucleotide positions might simplify genomic characterization of bacterial isolates particularly in complex samples with multiple strains and, the technique proposed herein, by exploring the variable positions represents a practical assay for genotyping the isolates of *P. aeruginosa* allowing extensive microbial population studies. Using *SNaPaer*, an informative and simple genotyping profile is obtained with a single reaction of multiplex PCR amplification and mini-sequencing.

## Materials and Methods

### *P. aeruginosa* Isolates and DNA Extraction

A total of 111 Portuguese isolates were obtained from sputum and blood samples of patients with pulmonary diseases (CF and patients admitted into intensive care units). The isolates were collected from three different Portuguese hospitals (Coimbra, Lisbon and Oporto) between 2009 and 2011. *P. aeruginosa* isolates were kept frozen at −80°C and cultured in Cetramide medium dishes before DNA extraction. Single colonies of each isolate were suspended in 5 mL of Lysogeny broth (LB) medium and grown overnight with 180 rpm agitation, at 37°C. DNA was extracted from cells according to a protocol suggested by Cheng and Jiang [31]. At the end, two DNA purification steps with ethanol (70%) were added to the protocol. Bacterial DNA was finally resuspended in 50 µl of ultrapure water and stored at −20°C.

### MLST Genotyping: Amplification and Sequencing

MLST was performed by using seven loci of housekeeping genes *acsA, aroE, guaA, nuoD, mutL, ppsA* and *trpE*, as previously described by Curran *et al*. [20]. The primers described by Curran *et al.* [20] were used and new ones were designed in cases of amplification problems. *Primer 3 (v 0.4.0)* was used for primer design (http://frodo.wi.mit.edu/) [32]. Subsequently, hairpin and primer-dimer secondary structures were avoided by using AutoDimer *v1* (http://www.cstl.nist.gov/strbase/AutoDimerHomepage/AutoDimerProgramHomepage.htm) [33]. The final set of primers employed for the amplification of MLST gene fragments is shown in Table S1. The PCR reaction was conducted in a final volume of 5 µL, containing 2.5 µL of 2x Qiagen multiplex PCR master mix (Qiagen), 1 µL of bacterial DNA (50–250 ng), 0.5 µL of primer mix (each one at 2 µM), 0.5 µL of Q-solution (Qiagen) and 0.5 µL of ultrapure water. PCR thermo-cycling conditions were: denaturation for 15 min at 95°C; 4 cycles with denaturation for 1 min at 95°C, primer annealing for 30 s at 68°C and extension for 2 min at 72°C; 4 cycles with denaturation for 1 min at 95°C, primer annealing for 30 s at 64°C and extension for 2 min at 72°C; 4 cycles with denaturation for 1 min at 95°C, primer annealing for 30 s at 61°C and extension for 2 min at 72°C; 23 cycles with denaturation for 1 min at 95°C, primer annealing for 30 s at 58°C and extension for 2 min at 72°C; and final extension for 10 min at 72°C. Amplicon sizes were confirmed after separation by polyacrylamide gel electrophoresis and standard silver-staining detection [34].

PCR products were purified with ExoSap-IT (*E. coli* exonuclease I and shrimp alkaline phosphatase; USB Corporation), according to the manufacturer instructions. Sequencing reactions were carried out using the ABI Big Dye terminator cycle sequencing ready reaction kit (Applied Biosystems). The sequencing reactions included incubation for 2 min at 96°C, followed by 35 cycles with denaturation for 15 s at 96°C, primer annealing for 9 s at 50°C, extension for 2 min at 60°C, and then 10 min at 60°C. Finally, the sequencing product was purified using SEPHADEX™ (Expansys) columns and a volume of 8.0 µL HiDi™ formamide (Applied Biosystems) was added before sequencing analysis in an ABI PRISM 3100 (Applied Biosystems) genetic analyzer.

### *SNaPaer* Assay

A set of 23 polymorphisms was selected from MLST genes for development of *SNaPaer* assay. Extension primers were designed with a forecasted Tm of approximately 60°C, at location near the target polymorphic position. A non-homologous tail was added at the 5′ end of extension primers for the separation by capillary electrophoresis. The final size of each primer ranged from 16 to 109 bp differing generally from each other by more than 3bp (Table 1). Hairpin and primer-dimer secondary structures were avoided by using AutoDimer *v1*. The assays were carried out in a final volume of 5 µL, containing 1.5 µL of PCR product (purified with ExoSap-IT, as described above), 1 µL of *SNaPaer* primer mix (each one at 1 µM), 1 µL of ABI Prism SNaPshot® Multiplex Kit (Applied Biosystems), and 1.5 µL of ultrapure water. The reaction was performed in 25 cycles at the following conditions: denaturation for 10 s at 96°C, primer annealing for 5 s at 55°C, and extension for 30 s at 60°C. Unincorporated ddNTPs were removed with 1 U of SAP (shrimp alkaline phosphatase; USB Corporation), after incubation for 1 h at 37°C and 15 min at 85°C. *SNaPaer* products (0.5 µL) were mixed with 9.0 µl of HiDi™ formamide (Applied Biosystems) and 0.5 µL of GeneScan-120 LIZ size standard (Applied Biosystems). Electrophoresis was performed on a 3130xl Genetic Analyzer (Applied Biosystems) using filter set E5 and the data analyzed with the software GeneMapper v 4.0 (Applied Biosystems).

**Table 1:** Primers used for single nucleotide polymorphism multiplex (*SNaPaer*).

Name*	Expected SNP§	Primer sequence (5′ to 3′)	Primerlenghtφ	Expectedpeakφ	Simpsonindex	Garza-Williamsonindex
**Ac7**	A/C/T	CCTACATCGTCTATGGYCCG	72	75–76	0.50	0.75
**Ac78**	A/G	TGACCCGCGTGGCGAA	26	33–34	0.38	0.67
**Ac336**	A/C/T	GCCCGGCTTCATCGC	66	68–69	0.50	0.75
**Ac387**	A/C/G	GCCGAGGTTGTCCACCAG	105	106–107	0.45	1.00
**A98**	C/G/T	GAACACCCTGATCCGCCT	47	50–52	0.41	1.00
**A264**	C/G/T	CGGTTGGCGATCAGCA	16	26–27	0.33	1.00
**A491**	C/G/T	ATGTAYGSCAAGGAACCGAC	93	95–96	0.40	1.00
**G6**	A/C/G/T	GGTTCCTCCAAGGTCCTGCT	69	70–71	0.51	1.00
**G49**	A/C/G/T	CCGATGGCCTTGTGCA	62	62–63	0.34	1.00
**G219**	C/T	TTGCGCTTCTCTTCCGG	39	46–47	0.33	0.67
**G264**	A/C/G/T	GGCCGCGCTTTCATCGAAGT	35	40–41	0.38	1.00
**M9**	A/G	GCCAGGCGCTTGATGAC	57	59–61	0.49	1.00
**M36**	C/G/T	GTGGAAAGCCACGTCGAA	78	80–82	0.50	0.67
**M204**	G/C/T	GCCTGCACCTGTGGGG	50	53–55	0.37	0.67
**M228**	A/C/G/T	CAGGTCCGGCTGGCTGCG	90	92–93	0.16	1.00
**N162**	C/T	CCAGTCCTGGCACAGTTTCAT	26	35–37	0.37	0.67
**N255**	C/G	GCCGGGATCAAGGTGCC	54	57–58	0.47	1.00
**N288**	C/G/T	GGTTCAGGATRCGGAAGAACTC	96	98–99	0.04	1.00
**P100**	A/C/G/T	GCTGGCCGATGGCACG	84	85–87	0.37	1.00
**P268**	A/C/G/T	GTCACCAACCGAGGAGGGCG	43	48–49	0.49	1.00
**T205**	C/G/T	TGGGGCGGGTGTCCGA	101	101–102	0.32	1.00
**T331**	A/C/T	ACGCGCTGCGGGCGAT	75	77–78	0.49	0.75
**T349**	A/G	TGCCGGCGGGYACKCT	109	107–109	0.46	0.67

* The name of primers is composed by MLST gene and polymorphic position.

§ Expected base on MLST profile.

φ Length of the primer plus tail of bases.

φ Expected position in the *SNaPaer* electropherogram.

*SNaPaer* assay was applied on a set of 111 *P. aeruginosa* isolates. A group of 20 isolates was initially tested in four independent experiments, being each experiment conducted more than two weeks apart; the remaining group of *P. aeruginosa* isolates was tested in duplicate with an interval of more than 3 weeks.

### Data and Statistical Analysis

*In silico* analyses were performed on MLST information from 1,177 online entries of *P. aeruginosa* (downloaded from http://pubmlst.org/paeruginosa/) grouped with MLST data of Portuguese bacteria. Network analysis was performed with the Network 4.6.1.0 program (www.fluxus-engineering.com/sharenet.htm) [35]. Minimum spanning trees were performed at MLST website (http://pubmlst.org/perl/mlstanalyse/mlstanalyse.pl?site=pubmlst&page=mst&referer=pubmlst.org). Statistical analysis was performed using Arlequin 3.1 software (http://cmpg.unibe.ch/software/arlequin3/) [36] and Microsoft Office Excel 2010 (Microsoft Corporation). Simpson's diversity index was used to determine the discriminatory power of individual SNPs and of the proposed multiplex strategy, according to the following formula:
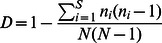
where, *n_i_* is the number of entities belonging to the *i*th type and *N* is the total number of entities in the dataset.

## Results

### Amplification of MLST Gene Fragments in a Single Multiplex Reaction

**Figure 1:**
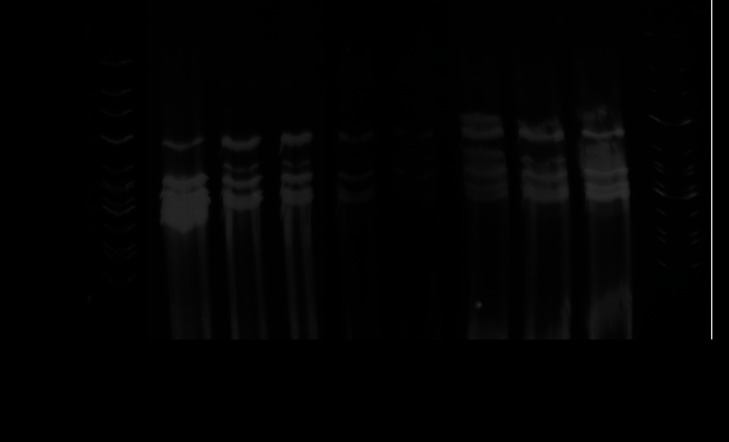
Polyacrylamide gel electrophoresis of MLST fragments amplified by *SNaPaer* multiplex PCR (L- Ladder; PA1 to PA8 - Different isolates of *Pseudomonas aeruginosa* **).**

Curran *et al.* [20] published a MLST genotyping strategy for *P. aeruginosa* based on singleplex amplifications of the housekeeping genes *acsA*, *aroE*, *guaA*, *mutL*, *nuoD, ppsA* and *trpE*. The primers described by Curran *et al.* [20] for the amplification of MLST targets were shown to be inadequate for multiplex amplification. Therefore, new primers were designed (see Table S1) and alternative touchdown PCR conditions (final temperature of 58°C) proposed for the amplification of seven MLST fragments in a single multiplex reaction. The newly designed genotyping protocol provided high reproducibility and hence enabled the detection and sequencing analysis of the gene fragments. The result of the multiplex amplification of a group of unrelated isolates of *P. aeruginosa* is shown in Figure 1.

### Selection of Polymorphisms for *SNaPaer* Assay

For SNP selection, MLST sequences available online (at http://pubmlst.org/paeruginosa/) were downloaded to a new database that included the genomic data of 30 unrelated *P. aeruginosa* strains from our collection. Key polymorphic positions located at MLST genes were selected based on its ability to discriminate *P. aeruginosa* strains. Non-polymorphic, redundant and low polymorphic positions were discarded. In addition, positions that are difficult to characterize on some MLST sequences were also excluded (a few examples of those ambiguous positions are shown in Figure S1). A last group of polymorphisms were removed due to difficulties in the design of primers. A set of 23 polymorphisms were finally selected on the seven housekeeping genes for inclusion in the *SNaPaer* assay, representing 0.01% of the total MLST nucleotides. The final analysis of the complete collection revealed that the target positions have a high discriminatory capacity within the collection of MLST profiles available online. Combining all selected markers, only a few online available sequence types were not differentiated (around 70% of the profiles were unique and the remaining were only shared by pairs of closely related sequence type; see complete list in Table S2). This set of 23 polymorphisms allows a theoretical number of possible combinations of more than 15 billion, which guarantees a high diversity of profiles that can be discriminated in *P. aeruginosa* employing *SNaPaer* assay (see Table 1 for the discriminatory power of each polymorphic position; only two polymorphisms showed diversity indexes below 0.3).

**Figure 2:**
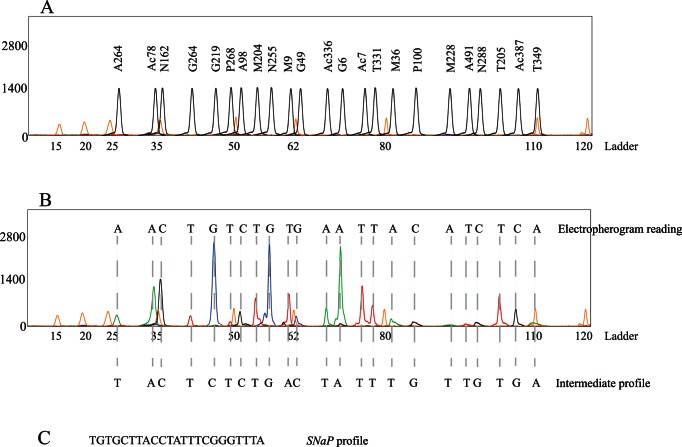
*SNaPaer* assay: A) position of each marker on the automated electropherogram (a total of 23 markers were included in *SNaPaer* assay); and B) example of *Pseudomonas aeruginosa* profile (peaks: orange – ladder; blue – guanine; black – cytosine; green – adenine; red – thymine); C) final *SNaP* profile of the isolate presented above obtained according to Table 1 (e.g. Ac7, Ac78, … T349) in order to facilitate comparison with MLST data. When markers are amplified using reverse primers (e.g. A264) the complementary base should be included in the *SNaP* profile.

Mini-sequencing primers ensured a distinct peak by automated capillary electrophoresis (Figure 2A); primers were individually tested facilitating the definition of the expected position on the electropherogram (Table 1). No primer interactions (namely primer dimers or hairpins) were observed when tested in multiplex. *SNaPaer* assay was further tested in the complete group of 111 clinical isolates of *P. aeruginosa* obtained from three distinct Portuguese Hospitals (located at Coimbra, Lisbon and Oporto). An example of a *SNaP* profile observed following the application of *SNaPaer* assay is shown in Figure 2; peaks with an intensity greater than 100 and in the expected electropherogram position (genomic fragments with the expected length) were considered valid. *SNaPaer* assay was highly reproducible and the same profile was obtained by distinct researchers testing the isolates in duplicate. The final *SNaP* profile was obtained following the *SNaPaer* data analysis; the markers were ordered in accordance with Table 1 facilitating the comparison of the genotypes with MLST data (genes are presented in alphabetic order). The analysis of our collection revealed 86 distinct *SNaP* profiles and a final discriminatory power of 0.9997 was observed in the Portuguese collection of *P. aeruginosa*; a similar value was observed when including the complete collection of sequence types available online (0.9993). Sequencing of MLST genes conducted in our group of isolates confirmed *SNaP* profiles, with a single exception corresponding to a new polymorphism in *aroE*.

### Population Analysis of Portuguese Clinical *P. aeruginosa*

**Figure 3:**
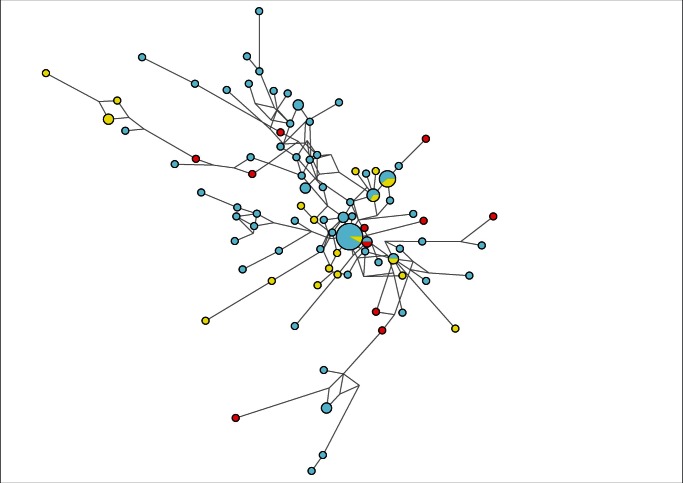
Network of the tested Portuguese strains (colors represent strains collected from three Hospitals: light blue – in Oporto, red - in Lisbon, yellow - in Coimbra; circles are representative of the proportion of profiles included in the network).

**Figure 4:**
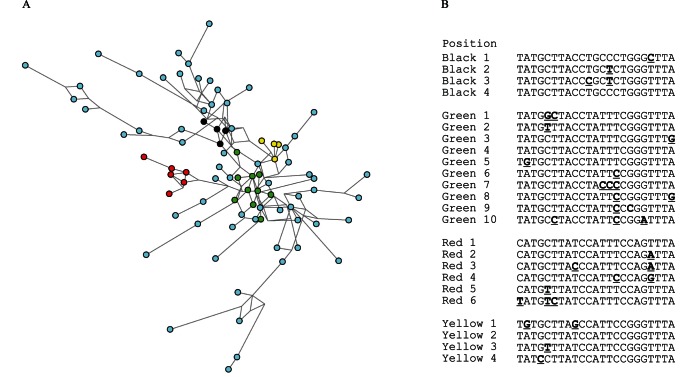
Network of Portuguese haplotypes of *Pseudomonas aeruginosa* showing microevolutionary events (difference in a single marker). (A) Black, green, red and yellow circles represent different sets of events shown in the population of *P. aeruginosa* and presenting the *SNaP* profiles shown in the table (B); light blue circles represent the remaining haplotypes.

The population analysis of *P. aeruginosa* isolates was conducted by employing Arlequin 3.1 software. The haplotype frequency revealed 72 novel and exclusive *SNaP* profiles not previously described at MLST database. The population profiles observed in clinical isolates from Oporto could not be distinguished from those collected in Lisbon or Coimbra; very few *SNaP* profiles (n = 3) were identified in populations from different hospitals (Figure 3). In contrast, isolates with similar *SNaP* profile were identified in distinct patients admitted to the same institution. Cases of microvariation (difference in a single marker) were frequently observed, particularly among the few isolates from our collection that came from the same patient along two years (Figure 4). Interestingly, this small group of patients monitored for two years was not only colonized by closely related strains (as shown in the network of Figure 4A) but also by strains with very distinct *SNaP* profile (Table S3).

We also analyzed in silico a set of 1,177 online MLST unique entries of *P. aeruginosa* (obtained from pubMLST at April 7, 2012) from different countries, between 1969 and 2011, in addition to the group of 111 Portuguese *P. aeruginosa* from this study. *SNaP* profiles found exclusively in Australia, Canada, China and Portugal were compared with the complete group of profiles by network analysis and application of minimum spanning tree; similar subpopulations were selected from profiles observed exclusively in two temporal intervals 1980–95 and 2005–11. No sub-groups of isolates could be observed in both spatial and temporal datasets (networks can be seen in Figures S2 and S3; minimum spanning tree resulted in similar distributions). Bacterial populations showed a widespread distribution along the networks, in concordance with Garza-Williamson (G-W) index value, which suggested absence of bottleneck events (Table 1).

## Discussion

The present study introduces an innovative methodology for genotyping *P. aeruginosa* based on the automatic analysis of 23 SNPs. *SNaPaer* assay represents a practical, reproducible, and sensitive alternative to MLST that allows the analysis of multiple polymorphisms in a single amplification and mini-sequencing reaction. Despite MLST being a widely employed methodology, the associated high costs and time consumption impose serious limitations to the study of large collections of bacterial isolates. Nonetheless, *P. aeruginosa* MLST database is one of the largest available online, which is justified by the enormous interest in this bacterium worldwide. *SNaPaer* assay uses a subset of MLST neutral genetic polymorphisms [37], responsible for synonym and non-synonym amino acid changes, that can be used to improve the diagnosis and surveillance of *P. aeruginosa* strains. A restriction fragment length polymorphism based assay had previously highlighted the advantage of SNP markers located in conserved genes for genotyping of clinical and environmental *P. aeruginosa* [28]. The newly proposed method allows *P. aeruginosa* genotyping in a single molecular reaction, in opposition to MLST which requires individual amplification and sequencing for each housekeeping gene. Thus, *SNaPaer* can cost six to seven times less than MLST and produces genotyping results in less than six hours. A weakness of *SNaPaer* is the loss of some genomic information compared to MLST particularly considering few point mutations, large deletions or insertions occasionally observed in the complete MLST genes; such differences were observed in a small group of closely relates isolates, as reported in the results section. Nevertheless, the theoretical number of possible combinations (more than 15 billion when employing 23 markers) guarantees a great potential for genetic diversity assessment.

*SNaPaer* genotyping may result in a low-cost test being particularly useful in cases of patients colonized or infected by multiple strains. Few alternatives have been recently developed for genome analysis of *P. aeruginosa,* reflecting the need for high throughput molecular approaches for epidemiological analysis. AT biochips [38] and high resolution melting curve-based SNP typing profile assay [39] represent good alternatives but are limited by biochip fabrication steps and expensive equipment required, and are prone to genotyping inaccuracies caused by background/non-specific fluorescence [40]. In 2011, Woo et al. [41] analyzed 35 *P. aeruginosa* MLST profiles and, aiming to promote cost-effectiveness of this strategy, suggested the reduction of seven to six housekeeping genes (removal of *trpE*) in *P. aeruginosa*. However, our study detected some informative polymorphic positions in *trpE*, and, therefore, we do not recommend such strategy. Instead *SNaPaer* assay uses less than 0.01% of the total length of MLST and presents a final discriminatory power of 0.9993. Moreover the limited number of polymorphisms tested with *SNaPaer* significantly reduces genotyping data analysis burden and the number of errors in contrast with the relative abundance of ambiguous results observed in MLST data.

We tested *SNaPaer* assay in a group of clinical isolates collected from distinct Portuguese hospitals. Point mutations were found to be the most abundant event in the diversification of these Portuguese samples and microevolution was frequently observed. Point mutations are also the major source of genetic variation observed among bacterial housekeeping genes [42]. The present analysis of Portuguese *SNaP* profiles corroborates the previously reported epidemic hypothesis of *P. aeruginosa* population [13], [28]; no spatial or temporal subpopulations could be identified. Multiple mutations were found in a small group of strains obtained from chronic colonized patients, suggesting either the reinfection by new strains or, in last instance, the occurrence of recombination or horizontal transfer events. Recombination events have been suggested and observed in few housekeeping genes, particularly among bacteria forced to adapt after clinical interventions [43].

In conclusion, *SNaPaer* assay represents a novel tool useful for identification and genotyping of *P. aeruginosa* strains. Moreover the primers used in the present work can be easily modified and extra markers accommodated when relevant for specific purposes/populations. This feature may be especially advantageous as the progress in the generation of sequence data [44] can substantially increase the number of target polymorphisms useful for *P. aeruginosa* genotyping. An online platform is presently under development and might become an alternative or a complement to the former successful MLST database. *SNaPaer* is also suitable for studying large collections of *P. aeruginosa* in a short period of time, due to its low cost high throughput and speed of analysis.

### Ethics Statement

The Ethics commissions of *Hospital de São João* and *Hospitais da Universidade de Coimbra* have approved the study. A written informed consent was provided by study participants and/or their legal guardians.

## Supporting Information

Figure S1 **Fragments of** ***mutL*** **and** ***ppsA*** **MLST genes.** Position 283 in *mutL* and 181 in *ppsA* present are ambiguous, thus these positions are difficult to define the final MLST profile.
(DOCX)

Figure S2 **Networks for** ***P. aeruginosa*** **profiles according to the place of isolation (data obtained from MLST website in addition to our collection).** MLST profiles from the online database were converted in *SNaP* profiles in order to design the networks: Australian (A), Canadian (B), Chinese (C) and Portuguese (D) haplotypes were marked dark blue; the remaining isolates are marked light blue.
(DOCX)

Figure S3 **Networks for** ***P. aeruginosa*** **profiles according to the isolation date (data obtained from the MLST website in addition to our collection).** MLST profiles from the online database were converted in *SNaP* profiles in order to design the network. Profiles detected in several years were excluded; isolates found from 1980 to 1995 were marked dark blue, while the isolates found from 2005 to 2011 were marked with light blue circles.
(DOCX)

Table S1 **Dideoxyoligonucleotide primers used for multilocus sequence typing of** ***Pseudomonas aeruginosa*** **.**
(DOCX)

Table S2 **Groups of online profiles that could not be separable by** ***SNaPaer*** **(allelic differences are marked blue).**
(XLSX)

Table S3 ***SNaP*** **profiles of** ***Pseudomonas aeruginosa*** **isolates obtained from the same patient.**
(DOCX)
