# Supplementary material for: SNaPaer: A Practical Single Nucleotide Polymorphism Multiplex Assay for Genotyping of Pseudomonas aeruginosa
Source: PLoS One. 2013 Jun 12;8(6):e66083. doi: 10.1371/journal.pone.0066083 (PMC3680407; doi:10.1371/journal.pone.0066083)
Supplement: Figure S1 — Fragments of mutL and ppsA MLST genes. Position 283 in mutL and 181 in ppsA present are ambiguous, thus these positions are difficult to define the final MLST profile. (DOCX) [file pone.0066083.s001.docx]

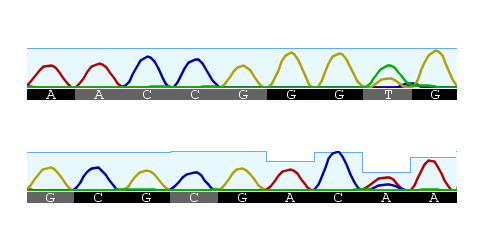

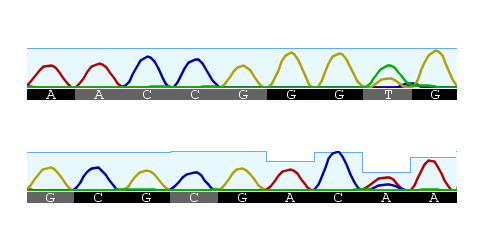


*ppsA*

*mutL*

174 175 176 177 178 179 180 181 182 bp

276 277 278 279 280 281 282 283 284 bp

Figure S1. Fragments of *mutL* and *ppsA* MLST genes. Position 283 in *mutL* and 181 in *ppsA* present are ambiguous, thus these positions are difficult to define the final MLST profile.
