# Supplementary material for: SNaPaer: A Practical Single Nucleotide Polymorphism Multiplex Assay for Genotyping of Pseudomonas aeruginosa
Source: PLoS One. 2013 Jun 12;8(6):e66083. doi: 10.1371/journal.pone.0066083 (PMC3680407; doi:10.1371/journal.pone.0066083)
Supplement: Figure S2 — Networks for P. aeruginosa profiles according to the place of isolation (data obtained from MLST website in addition to our collection). MLST profiles from the online database were converted in SNaP profiles in order to design the networks: Australian (A), Canadian (B), Chinese (C) and Portuguese (D) haplotypes were marked dark blue; the remaining isolates are marked light blue. (DOCX) [file pone.0066083.s002.docx]

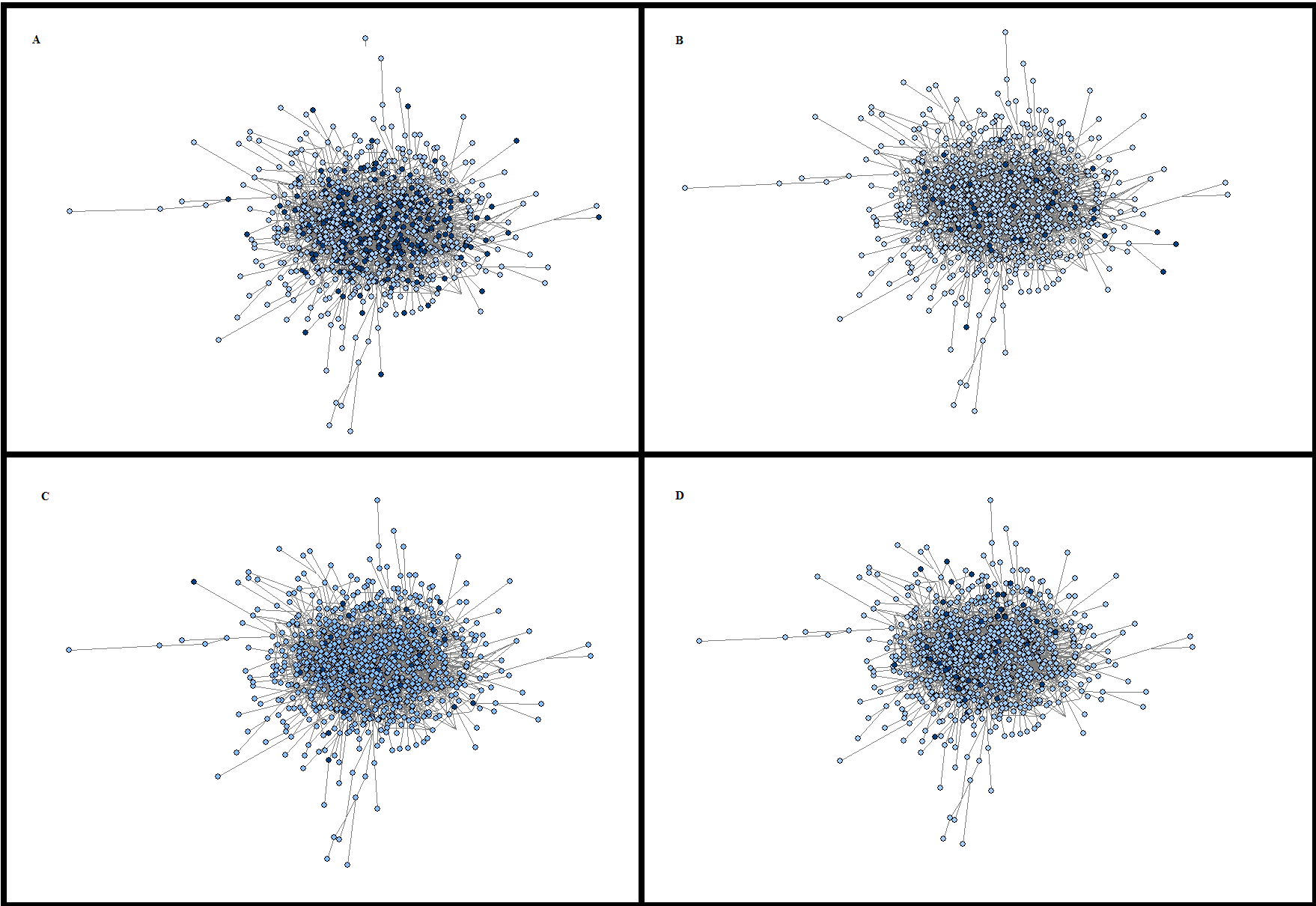


B

A

C

D

Figure S2. Networks for *P. aeruginosa* profiles according to the place of isolation (data obtained from MLST website in addition to our collection). MLST profiles from the online database were converted in *SNaP* profiles in order to design the networks: Australian (A), Canadian (B), Chinese (C) and Portuguese (D) haplotypes were marked dark blue; the remaining isolates are marked light blue.
