# Supplementary material for: SNaPaer: A Practical Single Nucleotide Polymorphism Multiplex Assay for Genotyping of Pseudomonas aeruginosa
Source: PLoS One. 2013 Jun 12;8(6):e66083. doi: 10.1371/journal.pone.0066083 (PMC3680407; doi:10.1371/journal.pone.0066083)
Supplement: Figure S3 — Networks for P. aeruginosa profiles according to the isolation date (data obtained from the MLST website in addition to our collection). MLST profiles from the online database were converted in SNaP profiles in order to design the network. Profiles detected in several years were excluded; isolates found from 1980 to 1995 were marked dark blue, while the isolates found from 2005 to 2011 were marked with light blue circles. (DOCX) [file pone.0066083.s003.docx]

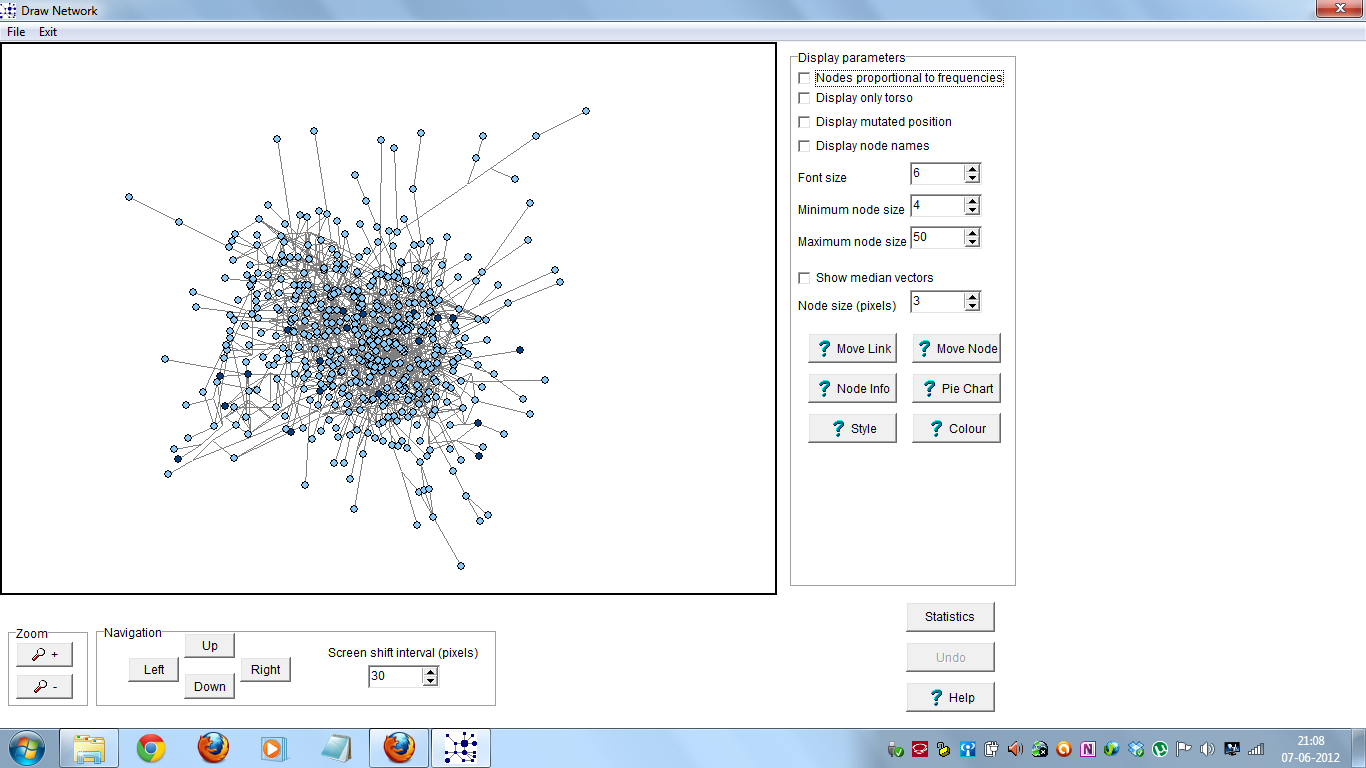


Figure S3. Networks for *P. aeruginosa* profiles according to the isolation date (data obtained from the MLST website in addition to our collection). MLST profiles from the online database were converted in *SNaP* profiles in order to design the network. Profiles detected in several years were excluded; isolates found from 1980 to 1995 were marked dark blue, while the isolates found from 2005 to 2011 were marked with light blue circles.
