# Supplementary material for: SNaPaer: A Practical Single Nucleotide Polymorphism Multiplex Assay for Genotyping of Pseudomonas aeruginosa
Source: PLoS One. 2013 Jun 12;8(6):e66083. doi: 10.1371/journal.pone.0066083 (PMC3680407; doi:10.1371/journal.pone.0066083)
Supplement: Table S1 — Dideoxyoligonucleotide primers used for multilocus sequence typing of Pseudomonas aeruginosa . (DOCX) [file pone.0066083.s004.docx]

**Table S1.** Dideoxyoligonucleotide primers used for multilocus sequence typing of *Pseudomonas aeruginosa*.

| Locus | Primer sequence (5’ to 3’) | | Fragment size (bp) |
| --- | --- | --- | --- |
| *acsA* | Forward^*^ | CTGGTGTACGCCTCGCTGAC | 837 |
|  | Reverse | TAGATGCCCTGCCCCTTGAT |  |
| *aroE* | Forward^*#^ | ACGATTTCCCCGGGTTC | 642 |
|  | Reverse | CGCGCCAGAGGAAGAAT |  |
| *guaA* | Forward^*§^ | CGGCCTCGACGTGTGGATGA | 844 |
|  | Reverse^§^ | GAACGCCTGGCTGGTCTTGTGGTA |  |
| *mutL* | Forward | AGCCTGGCAGGTGGAAAC | 634 |
|  | Reverse^*§^ | CAGGGTGCCATAGAGGAAGTC |  |
| *nuoD* | Forward^*§^ | ACCGCCACCCGTATCTG | 1080 |
|  | Reverse^§^ | TCTCGCCCATCTTGACCA |  |
| *ppsA* | Forward^*^ | GGGTAGCAAGGCGATCAAGATG | 1064 |
|  | Reverse | GGTTCTCTTCTTCCGGCTCGTAG |  |
| *trpE* | Forward^*^ | GCCGATCCCTCCGAGGAAAATG | 993 |
|  | Reverse^§^ | CCCGGCGCTTGTTGATGGTT |  |

^*^ Primers used for sequencing.

^§^ Primer obtained from Curran *et al*. [18]; all the other primers are from this work.

^#^ A second forward primer (5’ TATTCGGCAACCCCATCG 3’) was added on *aroE* to enhance gene amplification.
