# Supplementary material for: SNaPaer: A Practical Single Nucleotide Polymorphism Multiplex Assay for Genotyping of Pseudomonas aeruginosa
Source: PLoS One. 2013 Jun 12;8(6):e66083. doi: 10.1371/journal.pone.0066083 (PMC3680407; doi:10.1371/journal.pone.0066083)
Supplement: Table S3 — SNaP profiles of Pseudomonas aeruginosa isolates obtained from the same patient. (DOCX) [file pone.0066083.s006.docx]

|  | SNaP profile | Date of isolation |
| --- | --- | --- |
| Patient 1 | TATGCTTACCTGGGCTGGGCTTA  TATGCTTACCT**AAATC**GG**A**CTTA  TATGCTTACCTG**A**GCTGG**A**CTTA  TATGCTTACCTGGGCTGG**A**CTTA | March 2010  May 2010  September 2010  May 2011 |
| Patient 2 | TGTGGTTCCTCGAATCGGATTCG  TGTGGTTCCTCG**G**ATCGGATTCG  T**A**TG**C**TT**A**C**CTA**AATCGGA**C**T**TA**  TGT**C**GTTC**G**TCG**GG**TCGGA**C**T**T**G  T**A**TGCTT**A**C**CTA**AATCGGA**C**T**TA** | August 2009  October 2009  November 2009  November 2009  February 2011 |
| Patient 3 | CACGCTTACCCGAATCCGGCTCG  CA**TC**CTTACC**T**GA**GC**C**GCATCT**G | July 2009  October 2009 |
| Patient 4 | CACGCTTCTTCGGGCTGGATTTA  CA**T**G**T**TT**A**T**C**C**AAATCCA**A**C**TTA | October 2009  November 2009 |
| Patient 5 | TATGCTTATCCGGATCGGACTTA  TATGCTTA**C**C**T**G**AG**TCGGA**T**TTA | September 2010  May 2011 |
| Patient 6 | CACGCTTCTTCGGGCTGGATTTA **TGT**GCTT**AGC**CGAATCGGA**C**TTA  **TG**C**A**CTT**ACCT**GAATCGGATTT**G** | October 2009  April 2010  May 2011 |
| Patient 7 | TATGCTTACCTAAATCGGACTTA TAT**C**CTTACCT**G**A**G**TCGGA**T**TTA | August 2009  April 2011 |

Table S3. *SNaP* profiles of *Pseudomonas aeruginosa* isolates obtained from the same patient.
